# Sex and age-related differences in cerebral blood flow investigated using pseudo-continuous arterial spin labeling magnetic resonance imaging

**DOI:** 10.18632/aging.202673

**Published:** 2021-02-17

**Authors:** Joseph S. R. Alisch, Nikkita Khattar, Richard W. Kim, Luis E. Cortina, Abinand C. Rejimon, Wenshu Qian, Luigi Ferrucci, Susan M. Resnick, Richard G. Spencer, Mustapha Bouhrara

**Affiliations:** 1 Laboratory of Clinical Investigation, National Institute on Aging, National Institutes of Health, Baltimore, MD 20892, USA; 2 Laboratory Gerontology Branch, National Institute on Aging, National Institutes of Health, Baltimore, MD 20892, USA; 3 Laboratory of Behavioral Neuroscience, National Institute on Aging, National Institutes of Health, Baltimore, MD 20892, USA

**Keywords:** cerebral blood flow, aging, MRI, arterial spin labeling

## Abstract

Adequate cerebral blood flow (CBF) is essential to a healthy central nervous system (CNS). Previous work suggests that CBF differs between men and women, and declines with age and certain pathologies, but a highly controlled systematic study across a wide age range, and incorporating white matter (WM) regions, has not been undertaken. Here, we investigate age- and sex-related differences in CBF in gray matter (GM) and WM regions in a cohort (*N* = 80) of cognitively unimpaired individuals over a wide age range. In agreement with literature, we find that GM regions exhibited lower CBF with age. In contrast, WM regions exhibited higher CBF with age in various cerebral regions. We attribute this new finding to increased oligodendrocyte metabolism to maintain myelin homeostasis in the setting of increased myelin turnover with age. Further, consistent with prior studies, we found that CBF was higher in women than in men in all brain structures investigated. Our work provides new insights into the effects of age and sex on CBF. In addition, our results provide reference CBF values for the standard ASL protocol recommended by the ISMRM Perfusion Study Group and the European ASL in Dementia consortium. Thus, these results provide a foundation for further investigations of CNS perfusion in a variety of settings, including aging, cerebrovascular diseases, and dementias.

## INTRODUCTION

Age is the main risk factor for neurodegeneration in the central nervous system (CNS) and concomitant cognitive and functional impairments. Cerebral blood flow (CBF), the rate of arterial blood flow through the capillary bed in cerebral tissue, is the main determinant of oxygen and substrate delivery as well as clearance of metabolic by-products. Several lines of research suggest that CBF is a critical biomarker affected by normal aging as well as a myriad of neurodegenerative diseases [1]. Indeed, mounting evidence suggests that perfusion plays an important role in the progression of many neurodegenerative processes, including Alzheimer’s disease [2]. Therefore, changes in CBF with age may be causally linked to age-associated pathology. Characterizing the changes in CBF that occur with normal aging in the absence of clinically detectable pathology is of further importance to distinguish this from specific pathologic effects.

CBF is conventionally measured using techniques such as positron-emission tomography, single-photon emission computerized tomography, or computed tomography. These methods require injection of contrast agents or radioactive tracers which require exposure to ionizing radiation or may cause nephrotoxicity. However, arterial spin labeling (ASL) magnetic resonance imaging (MRI) techniques permit whole-brain CBF mapping within a few minutes. Briefly, ASL makes use of magnetic labeling of arterial water protons by radiofrequency pulses [3], upstream of the volume of interest. It is a difference technique, requiring subtraction of a labeled image from a control, non-labeled, image. The difference between these two images corresponds to the effect of the labeling procedure, resulting in a perfusion-weighted image that can be combined with an additional proton density-weighted image to derive a CBF map [4].

ASL has been widely used to investigate CBF in various neurological disorders and to characterize age- and sex-effects. While there is consistent evidence for an overall decrease in CBF with age, conclusions have been mixed regarding the effects of age and sex on regional CBF. Indeed, while some studies have shown that cortical CBF decreases with age, other investigations have indicated the opposite trend or found no correlation with age [5–13]. Further, Parkes and colleagues [14] have shown that WM CBF increases with age using continuous ASL, while Liu and colleagues’ study suggested that WM CBF decreases with age in women using pseudo-continuous ASL (pCASL) [8]. These discrepancies are likely due to limited cohort sizes as well as the technical challenges of implementing truly quantitative CBF determination using ASL. In addition, most previous studies focused on brain GM, with little information available regarding CBF in cerebral WM. This is likely due to the lower CBF values in WM along with the high sensitivity to noise of CBF values derived from ASL especially given that it is a subtraction technique for an effect of limited dynamic range. With the development of new postprocessing analysis techniques, including the nonlocal estimation of multispectral magnitudes (NESMA)-ASL filter [15], more accurate analysis of CBF in WM has become feasible.

Our main goal in the present work is to characterize the dependence of regional CBF on age and sex in critical GM and WM brain regions, and to provide reference CBF values using the standardized ASL protocol recommended by the ISMRM Perfusion Study Group and the European ASL in Dementia consortium [4]. Our investigation was conducted on a cohort (*N* = 80) of cognitively unimpaired participants spanning the wide age range of 22 to 88 years.

## RESULTS

### Visualization of cerebral blood flow maps at different ages

**Figure 1:**
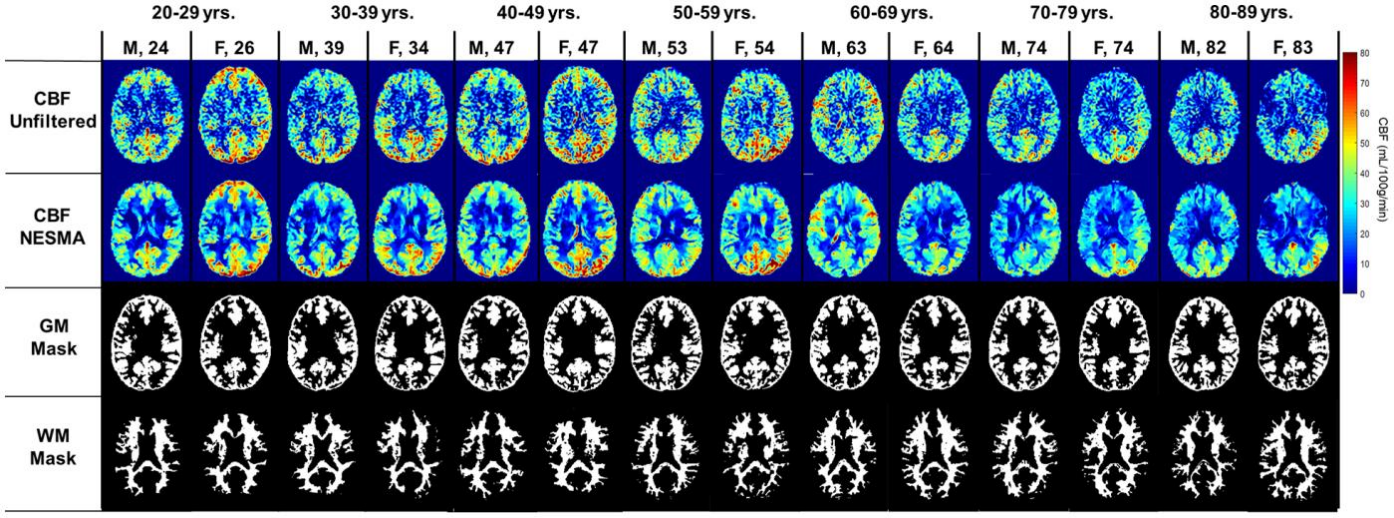
**Cerebral blood flow (CBF) maps derived from pCASL imaging datasets with or without the NESMA-ASL filter.** Corresponding GM and WM masks generated using FSL-FASL are also displayed. Results are shown for fourteen representative male and female participants within each age decade.

Figure 1 shows a representative axial slice of derived CBF maps from brains of male and female participants within each age decade of our cohort. CBF maps derived with (first row) or without (second row) NESMA filtering of the ASL images are displayed. It is readily seen that, as expected, NESMA substantially reduces random variation in derived CBF maps. Further, visual inspection indicates that CBF varies with age, with the most pronounced variations occurring in the GM regions; this is clearly visible from the CBF maps derived after NESMA filtering. It is also observed that older participants have lower GM CBF as compared to young participants within the third, fourth, and fifth age decades. However, CBF varies minimally with age in the WM regions.

### Effects of age and sex on CBF

**Figure 2:**
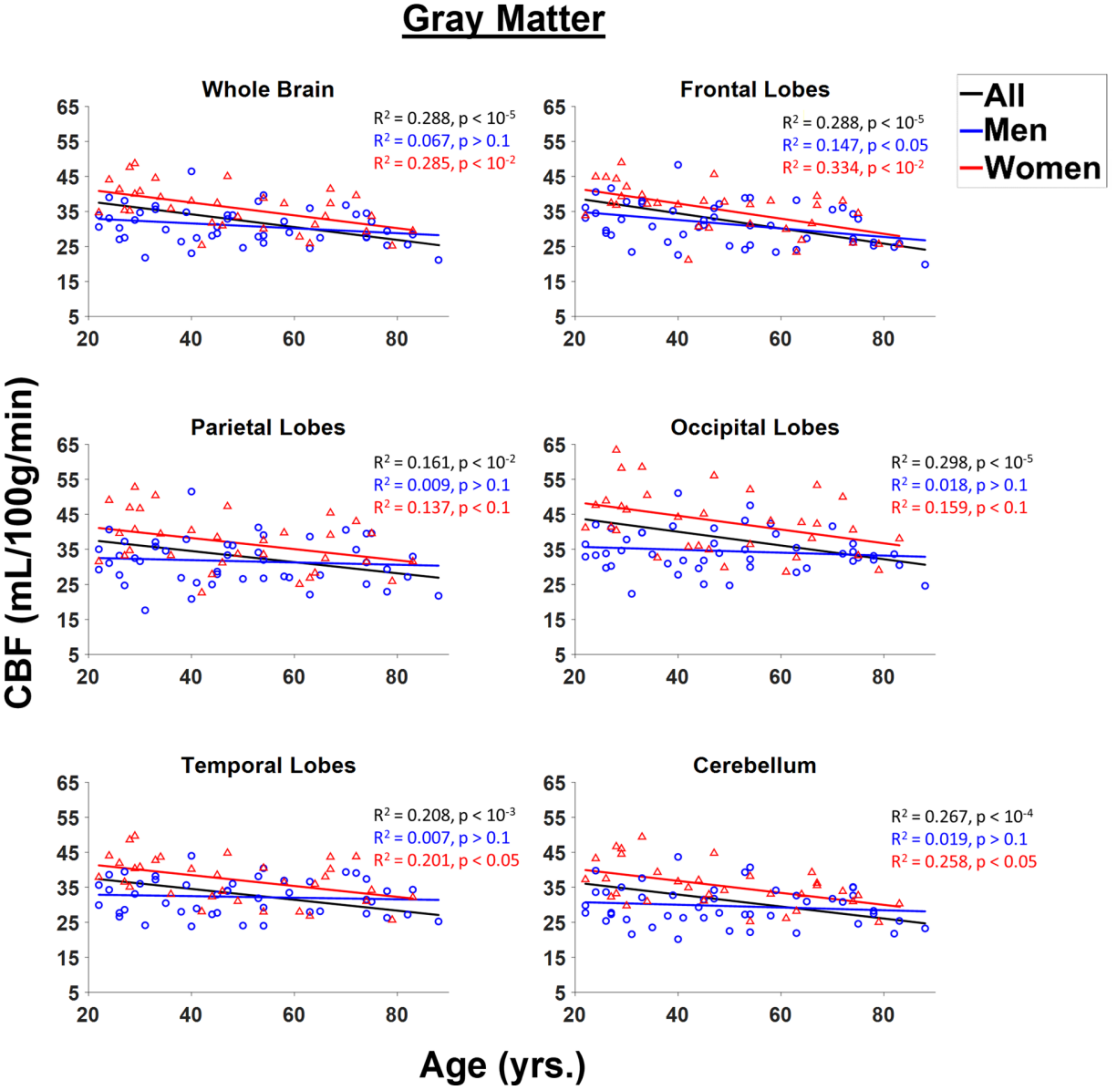
**Regressions of NESMA-CBF with age and sex shown for the six gray matter (GM) regions investigated.** For each structure, the coefficient of determination, *R*^2^, and *p*-value, obtained after FDR correction, are reported. Most regions investigated showed linearly decreasing CBF with age.

**Figure 3:**
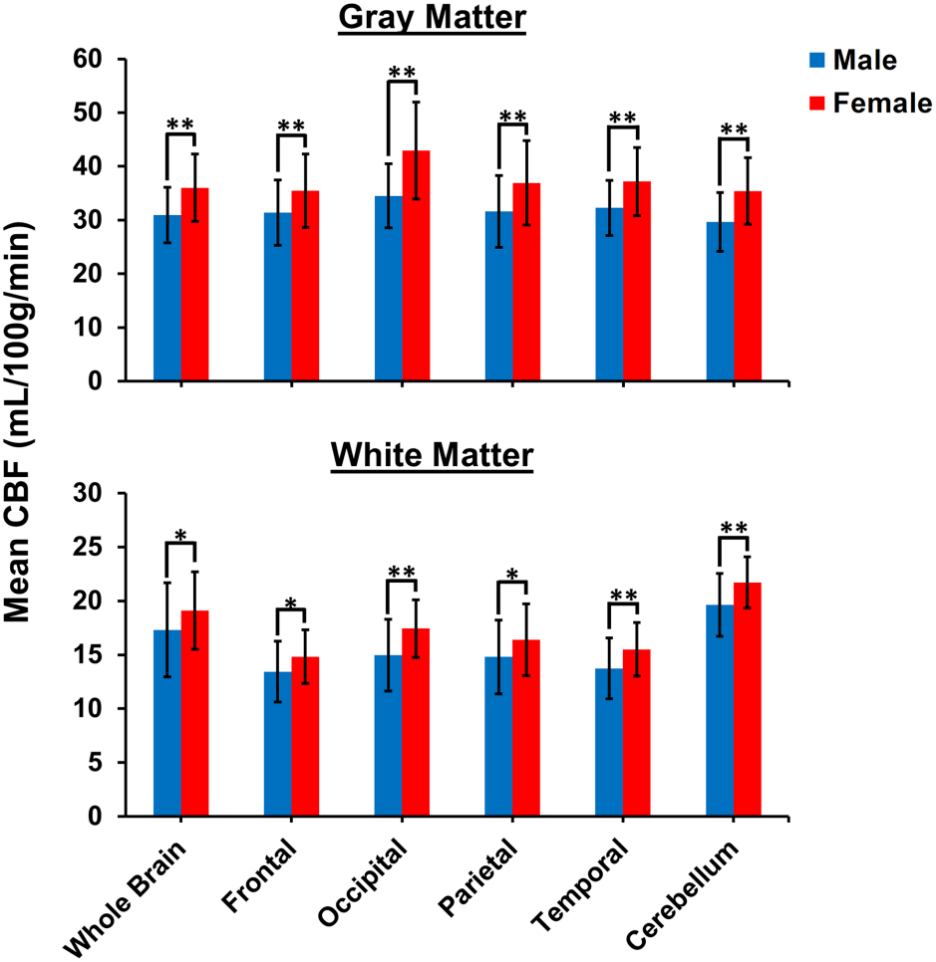
**Comparison of mean CBF values obtained from NESMA-filtered ASL images for men and women in the indicated GM and WM regions.** Mean CBF values for women are overall significantly greater than mean CBF values for men.

Figure 2 shows linear relationships between CBF measurements using NESMA-filtered ASL images and age in the GM ROIs for men and women separately, and for all participants taken together. GM CBF was found to decrease with age, with regional variation among regions (Figure 2). Statistical analysis of all participants showed that all brain structures exhibited significant (*p* < 0.05) decreases of CBF with age except for the GM within the parietal lobes (Table 1). In addition, the most rapid decline in CBF with respect to age was found in the frontal lobes, while the slowest decline was in the temporal lobes (Table 1). For all GM ROIs, women exhibited significantly higher CBF values as compared to men (Figure 3). For women, all GM ROIs showed significant decreases in CBF with age (Table 2). For men, the frontal lobes showed significant decreases with respect to age, while the whole brain showed a non-significant trend towards a decrease in CBF with age (Table 2). Furthermore, women exhibited steeper slopes in the decline in CBF with age as compared to men for all GM ROIs with, for both men and women, the frontal lobes exhibiting the steepest decline. The rate with respect to age for the decline in CBF for women was found to be significantly different from the rate for men in the cerebellum, the whole brain, and the occipital and temporal lobes. We note that the interactions between age and sex were not significant in any ROI after FDR correction. Finally, although qualitative CBF results derived from NESMA-filtered and from unfiltered ASL images were similar in GM regions, the finding of a significant dependence on age was greatly strengthened with NESMA filtering in several brain structures including the occipital and parietal lobes, and the cerebellum (Table 1). This is consistent with the effect of the NESMA filter to decrease image noise, and hence variance of parameter estimation [15].

**Table 1:** Significance (*p*-value) and slope of the variation in CBF as a function of age and sex for the linear regression analysis for each GM and WM brain region studied.

	**NESMA filtered**									**Unfiltered**							
	**Gray matter (GM)**				**White matter (WM)**					**Gray matter (GM)**				**White matter (WM)**			
	**Age**		**Sex**		**Age**		**Sex**			**Age**		**Sex**		**Age**		**Sex**	
	**p-value**	**Slope**	**p-value**	**Slope**	**p-value**	**Slope**	**p-value**	**Slope**		**p-value**	**Slope**	**p-value**	**Slope**	**p-value**	**Slope**	**p-value**	**Slope**
**Whole Brain**	**< 0.01**	-0.11	**< 0.01**	-4.95	**< 0.01**	0.08	**< 0.05**	-1.90		**< 0.01**	-0.11	**< 0.01**	-4.92	**< 0.01**	0.07	**< 0.1**	-1.41
**Frontal Lobes**	**< 0.01**	-0.16	**< 0.01**	-3.85	**< 0.05**	0.04	**< 0.05**	-1.44		**< 0.01**	-0.15	**< 0.01**	-3.85	**< 0.05**	0.02	> 0.1	-1.04
**Occipital Lobes**	**< 0.05**	-0.10	**< 0.01**	-8.32	**< 0.01**	0.06	**< 0.01**	-2.56		**< 0.1**	-0.10	**< 0.01**	-8.35	**< 0.01**	0.04	**< 0.01**	-1.96
**Parietal Lobes**	**< 0.1**	-0.08	**< 0.01**	-5.20	**< 0.01**	0.06	**< 0.05**	-1.66		> 0.1	-0.08	**< 0.01**	-5.32	**< 0.05**	0.04	**< 0.05**	-1.20
**Temporal lobes**	**< 0.05**	-0.07	**< 0.01**	-4.82	**< 0.01**	0.05	**< 0.01**	-1.84		**< 0.05**	-0.07	**< 0.01**	-4.82	**< 0.05**	0.03	**< 0.01**	-1.34
**Cerebellum**	**< 0.05**	-0.09	**< 0.01**	-5.64	**< 0.05**	0.04	**< 0.01**	-2.12		**< 0.1**	-0.10	**< 0.01**	-5.38	**< 0.05**	0.02	**< 0.01**	-1.85

All *p*-values presented are obtained after FDR correction. Results are shown for both CBF maps derived with or without the NESMA filtering of the ASL images.

**Table 2:** Significance (*p*-value) and slope of the variation in NESMA-CBF as a function of age and sex for the GM and WM regions studied, for men and for women.

	**Men**					**Women**			
	**GM**		**WM**			**GM**		**WM**	
	**p-value**	**Slope**	**p-value**	**Slope**		**p-value**	**Slope**	**p-value**	**Slope**
**Whole Brain**	**< 0.1**	-0.07	**< 0.01**	0.11		**< 0.01**	-0.18	> 0.1	0.05
**Frontal Lobes**	**< 0.01**	-0.12	**< 0.05**	0.05		**< 0.01**	-0.21	> 0.1	0.03
**Occipital Lobes**	> 0.1	-0.04	**< 0.05**	0.06		**< 0.05**	-0.20	**< 0.1**	0.05
**Parietal Lobes**	> 0.1	-0.03	**< 0.01**	0.07		**< 0.05**	-0.16	> 0.1	0.04
**Temporal Lobes**	> 0.1	-0.02	**< 0.01**	0.05		**< 0.01**	-0.16	> 0.1	0.03
**Cerebellum**	> 0.1	-0.04	> 0.1	0.02		**< 0.01**	-0.17	**< 0.05**	0.04

All *p*-values presented are obtained after FDR correction.

**Figure 4:**
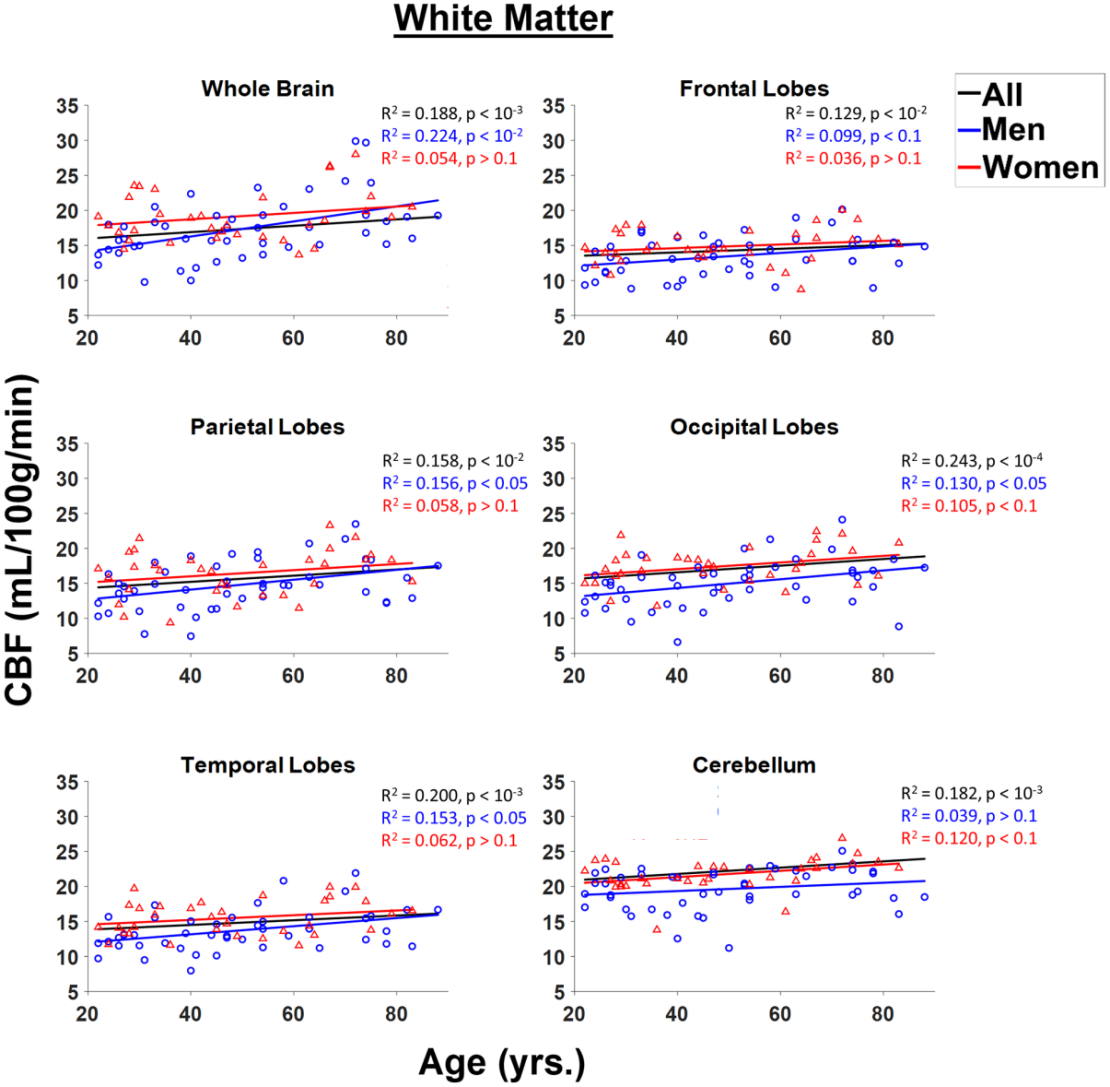
**Regressions of CBF values obtained from NESMA-filtered ASL images with age and sex shown for the six white matter (WM) regions investigated.** For each structure, the coefficient of determination, *R*^2^, and *p*-value, obtained after FDR correction, are reported. Most regions investigated showed linearly increasing CBF with age. This trend is more pronounced for men (blue) as compared to women (red).

Figure 4 shows linear relationships between CBF measurements using NESMA-filtered ASL images and age in the WM ROIs for men and women separately, and for all participants taken together. WM CBF increased slightly but significantly with age, both for the whole brain and across all examined regions (Figure 4, Table 1). Further, for all WM ROIs, women exhibited significantly higher CBF values as compared to men (Figure 3). Women exhibited a steeper and more statistically significant increase with respect to age in the cerebellum, and a smaller rate of increase with respect to age as compared to men in the occipital lobes that approached significance (Figure 3, Table 2). For men, increases in WM CBF as a function of age were seen in all brain regions investigated except in the cerebellum (Table 2). Furthermore, men exhibited relatively larger rates with respect to age for the decline in CBF as compared to women for all ROIs studied except in the cerebellum. The interactions between age and sex were not significant in all ROIs after FDR correction. Table 3 provides a detailed summary of GM and WM CBF values. Finally, results of CBF derived from NESMA-filtered ASL images as compared to those derived from unfiltered ASL images were substantially different in WM regions. Indeed, the regression coefficients calculated from CBF maps derived from filtered ASL images exhibited, overall, higher values in most WM regions examined (Table 1). Here as well, significance with age was strengthened with NESMA-filtering in most ROIs, including within the parietal lobes and temporal lobes.

**Table 3:** The mean and standard deviation (SD) of CBF values, derived from NESMA filtered ASL images, for all participants as well as for men or women, for each ROI and for each age interval as well as across the entire age range.

**Mean** ± **SD CBF values (mL/100g/min)**										
			**20–29 yrs.**	**30–39 yrs.**	**40–49 yrs.**	**50–59 yrs.**	**60–69 yrs.**	**70–79 yrs.**	**80–89 yrs.**	**All**
**All Participants**	**GM**	Whole Brain	36.4 ± 6.40	34.5 ± 6.45	32.9 ± 6.39	31.9 ± 5.50	31.6 ± 5.77	31.2 ± 4.58	26.1 ± 3.71	33.0 ± 6.17
		Frontal Lobes	37.3 ± 6.14	34.9 ± 5.76	33.7 ± 7.14	31.2 ± 6.15	30.8 ± 6.05	30.6 ± 4.92	24.0 ± 2.80	33.0 ± 6.66
		Occipital Lobes	41.3 ± 9.41	39.4 ± 9.92	36.9 ± 8.56	38.7 ± 8.02	36.7 ± 8.26	35.7 ± 5.75	31.7 ± 5.65	37.9 ± 8.43
		Parietal Lobes	36.4 ± 7.66	35.5 ± 8.85	32.9 ± 8.57	33.1 ± 5.68	31.7 ± 7.74	33.5 ± 6.87	28.3 ± 5.04	33.8 ± 7.59
		Temporal Lobes	36.9 ± 6.76	35.4 ± 6.10	33.5 ± 5.98	33.0 ± 6.05	33.9 ± 6.28	33.3 ± 5.66	29.7 ± 4.21	34.2 ± 6.14
		Cerebellum	35.3 ± 6.82	31.7 ± 7.98	32.6 ± 6.26	30.6 ± 6.74	31.5 ± 5.41	30.6 ± 3.59	25.1 ± 3.71	32.0 ± 6.43
	**WM**	Whole Brain	16.6 ± 2.92	17.2 ± 4.35	16.6 ± 3.10	17.3 ± 3.38	19.2 ± 4.84	22.1 ± 4.97	18.7 ± 1.90	18.0 ± 4.13
		Frontal Lobes	12.8 ± 2.25	14.3 ± 3.16	13.7 ± 2.13	13.2 ± 2.55	14.6 ± 3.44	15.7 ± 3.27	14.4 ± 1.36	14.0 ± 2.77
		Occipital Lobes	15.0 ± 2.67	14.7 ± 3.47	15.1 ± 3.29	16.9 ± 2.59	17.4 ± 3.31	17.4 ± 3.34	16.3 ± 5.19	15.9 ± 3.29
		Parietal Lobes	14.4 ± 2.94	14.4 ± 4.14	14.4 ± 3.40	15.1 ± 2.32	17.4 ± 3.59	17.8 ± 3.56	15.3 ± 1.91	15.4 ± 3.45
		Temporal Lobes	13.5 ± 2.32	13.8 ± 2.81	13.7 ± 2.63	14.8 ± 2.97	15.1 ± 3.10	16.1 ± 3.10	15.3 ± 2.58	14.4 ± 2.82
		Cerebellum	20.8 ± 1.95	18.7 ± 2.99	19.7 ± 3.00	20.0 ± 3.35	21.4 ± 2.44	22.8 ± 2.26	18.8 ± 2.73	20.4 ± 2.87
**Men**	**GM**	Whole Brain	32.4 ± 4.17	31.3 ± 5.58	31.6 ± 6.57	30.6 ± 5.49	29.3 ± 5.92	30.9 ± 4.04	25.0 ± 3.65	30.9 ± 5.18
		Frontal Lobes	33.9 ± 4.84	32.6 ± 5.94	33.3 ± 7.06	29.7 ± 6.34	29.8 ± 7.41	30.4 ± 4.65	23.5 ± 3.23	31.3 ± 6.05
		Occipital Lobes	34.9 ± 4.29	35.1 ± 6.79	34.3 ± 7.87	36.8 ± 7.6	31.1 ± 3.76	34.4 ± 3.27	29.5 ± 4.59	34.5 ± 5.95
		Parietal Lobes	32.3 ± 4.91	31.6 ± 7.24	31.7 ± 9.10	31.7 ± 5.91	29.4 ± 8.42	32.9 ± 6.84	27.2 ± 5.60	31.6 ± 6.65
		Temporal Lobes	32.6 ± 4.72	32.8 ± 5.32	32.0 ± 5.97	32.2 ± 6.20	30.9 ± 4.94	33.2 ± 5.06	28.9 ± 4.78	32.2 ± 5.16
		Cerebellum	31.1 ± 4.66	28.6 ± 5.71	30.1 ± 6.51	30.0 ± 7.17	28.5 ± 5.75	30.7 ± 3.70	23.4 ± 1.82	29.6 ± 5.50
	**WM**	Whole Brain	15.1 ± 1.88	15.5 ± 3.84	15.9 ± 3.97	17.1 ± 3.59	18.5 ± 4.06	22.1 ± 5.64	18.1 ± 1.83	17.3 ± 4.35
		Frontal Lobes	11.8 ± 1.87	13.2 ± 3.34	13.2 ± 2.69	12.8 ± 2.59	15.9 ± 3.02	14.8 ± 3.47	14.2 ± 1.58	13.4 ± 2.83
		Occipital Lobes	13.6 ± 1.84	13.7 ± 3.34	13.5 ± 3.37	16.8 ± 2.78	15.2 ± 2.96	17.1 ± 3.53	14.8 ± 5.22	14.9 ± 3.32
		Parietal Lobes	13.2 ± 1.96	13.4 ± 3.53	13.8 ± 4.12	15.3 ± 2.4	17.1 ± 3.13	17.1 ± 4.13	15.4 ± 2.34	14.8 ± 3.42
		Temporal Lobes	12.5 ± 1.59	12.9 ± 2.7	12.4 ± 2.55	14.8 ± 3.08	13.5 ± 2.21	15.7 ± 3.42	14.9 ± 3.01	13.7 ± 2.83
		Cerebellum	19.9 ± 1.78	18.6 ± 3.02	18.3 ± 3.22	19.5 ± 3.84	20.8 ± 1.75	21.9 ± 2.04	17.6 ± 1.36	19.6 ± 2.90
**Women**	**GM**	Whole Brain	40.8 ± 5.59	40.0 ± 3.63	34.5 ± 6.27	35.3 ± 4.68	32.8 ± 5.86	31.8 ± 6.16	29.4 ± n/a	36.0 ± 6.28
		Frontal Lobes	41.2 ± 5.18	39.7 ± 2.36	34.3 ± 7.77	35.4 ± 3.55	31.2 ± 5.99	31.0 ± 6.16	25.4 ± n/a	35.4 ± 6.9
		Occipital Lobes	48.5 ± 8.34	45.8 ± 12.9	40.2 ± 8.80	43.8 ± 7.85	39.5 ± 8.68	38.2 ± 9.16	38.0 ± n/a	42.7 ± 9.06
		Parietal Lobes	41.0 ± 7.84	43.4 ± 9.01	34.4 ± 8.27	36.9 ± 3.16	32.8 ± 7.94	34.9 ± 7.75	31.5 ± n/a	36.8 ± 7.96
		Temporal Lobes	41.7 ± 5.39	38.8 ± 5.14	35.5 ± 5.85	34.9 ± 6.37	35.4 ± 6.73	33.6 ± 7.60	32.1 ± n/a	36.9 ± 6.33
		Cerebellum	40.0 5.75	37.3 ± 9.08	35.9 ± 4.40	32.1 ± 6.52	33.0 ± 5.03	30.6 ± 3.91	30.2 ± n/a	35.4 ± 6.19
	**WM**	Whole Brain	18.2 ± 3.07	20.3 ± 3.76	17.5 ± 1.17	17.8 ± 3.40	19.5 ± 5.53	22.2 ± 4.04	20.4 ± n/a	19.1 ± 3.60
		Frontal Lobes	13.9 ± 2.21	16.8 ± 1.73	14.3 ± 0.98	14.2 ± 2.65	13.9 ± 3.72	17.6 ± 2.12	15.1 ± n/a	14.8 ± 2.53
		Occipital Lobes	16.4 ± 2.76	15.8 ± 3.71	17.2 ± 1.62	17.2 ± 2.54	18.5 ± 3.11	18.1 ± 3.33	20.7 ± n/a	17.4 ± 2.70
		Parietal Lobes	15.7 ± 3.42	16.1 ± 6.14	15.3 ± 2.21	14.7 ± 2.49	17.6 ± 4.07	19.3 ± 1.53	15.2 ± n/a	16.3 ± 3.38
		Temporal Lobes	14.7 ± 2.56	14.8 ± 2.78	15.4 ± 1.71	14.9 ± 3.29	15.9 ± 3.36	16.9 ± 2.60	16.5 ± n/a	15.4 ± 2.50
		Cerebellum	21.7 ± 1.74	18.8 ± 3.38	21.6 ± 1.06	21.3 ± 1.08	21.6 ± 2.84	24.5 ±1.66	22.6 ± n/a	21.7 ± 2.37

## DISCUSSION

In this cross-sectional study of a cohort of cognitively unimpaired participants, we investigated CBF as a function of age and sex within twelve cortical and white matter cerebral structures. Our results indicate that CBF decreases with age in all GM regions investigated, consistent with previous studies [5–8, 13, 16]. This age-related reduction in CBF may reflect decreased cerebral metabolic demand, decreased neuronal firing, decreased dendritic synaptic density, as well as cerebrovascular deterioration [16–21]. Indeed, aging is accompanied by changes in brain structure which likely lead to a decreased metabolic demand while also rendering it particularly vulnerable to neurodegenerative processes [22].

There is increasing evidence of an association between brain hypoperfusion and dementia [23, 24], so that characterizing normative age-related changes in CBF may represent a fundamental step towards differentiating between normative aging and pathology. Several previous studies have reported a decrease in cortical CBF with age, while others have found no trend or an increase [5–12]. Comparison of these results with the present ones is difficult due to differences in cohort size, non-standardized methodology, and analysis of different brain regions. Our work is distinguished by the use of a large cohort and a modern, sensitive, ASL MRI sequence, pCASL, incorporation of NESMA filtering, and optimized experimental parameters. Indeed, our advanced postprocessing analysis [15] allowed us to provide results for WM as well as GM regions.

Our findings of statistically significant increases in CBF with age in all WM regions investigated agrees with previous studies [11, 12, 14]. However, the literature regarding age-related differences in CBF in WM regions is limited and results are sparse. This is likely due to the high degree of noise sensitivity of CBF values derived from ASL, especially in WM with its inherently low CBF values. We provisionally attribute the observed increase in WM CBF with age to increased oligodendrocyte metabolic demand for production and maintenance of myelin homeostasis [25, 26]. Indeed, studies have shown that brain undergoes rapid myelin loss after the fourth age decade [27–31]. However, further studies, especially longitudinal studies, are required to elucidate the mechanisms underlying CBF changes in WM.

We note that re-analysis of our data using unfiltered CBF maps showed trends similar to the results presented for filtered maps (Table 1). Thus, the trends of increasing CBF with age in WM observed in this work are independent of filtering. However, with the improvement in parameter estimation derived from use of filtering, the power of the analysis is greatly increased. Our results must be interpreted with caution. Indeed, it has been shown that a high signal-to-noise ratio, achieved through a large number of signal averages, is required to detect perfusion signal in deep white matter regions [32]. Furthermore, the ASL protocol used in our study is optimized for GM CBF. ASL studies using multiple post-labeling delays are required for further validation [33].

The details of the linear relationships between CBF and age, including their slopes and statistical significance, will exhibit some variability as a function of sampling density within age groups, range of ages incorporated, and consistency of data [34]. In addition, the choice of a linear regression model, while conventional and consistent with our visual inspection of the results, is best considered as an expedient to model the data, without the implication that it is based on the biology of underlying physiologic processes. Nonlinear models may serve equally well as data descriptors. Indeed, in a very large cohort of youth participants, it has been demonstrated that the relationships between CBF and age are best described by nonlinear trends [35]. However, the present analysis provides a basic description of the variation of CBF with age in adulthood. Moreover, at ages younger or older than our sample, the trends with respect to age may deviate substantially from the indicated regression results. The fundamental physiology of these extremes of age may differ from that within the age range we are investigating.

In further agreement with the literature, our results indicated that women exhibit significantly higher CBF values than men [8, 14, 36–41] in most WM and GM structures investigated. This may be attributed to several factors including sex differences in heart rate, blood pressure, and hematocrit, all of which may modulate CBF [41–44]. Indeed, it has been shown that CBF quantification from ASL using a fixed hematocrit of 43.5%, as generally assumed, may lead to bias in derived CBF values particularly in non-European or female subjects, so that individually measured hematocrit should be considered to improve determination of CBF [43]. In addition, recent studies have shown that women have higher myelin content than men [25, 26]; this may also explain differences in CBF in WM, with these myelinated regions exhibiting increased metabolic demand to maintain myelin homeostasis. Differences in sex hormones may also contribute to the differences in CBF observed between men and women. Indeed, studies have shown that estrogen and testosterone have different effects on CBF [8, 41, 45, 46]; overall, estrogen decreases cerebral vascular tone and increases CBF by enhancing endothelial-derived nitric oxide and prostacyclin pathways, while testosterone increases cerebral artery tone. From this perspective, declining estrogen levels following menopause [47, 48] could further contribute to the observation that women exhibit statistically significant decreases in CBF compared to men found here and in other studies [8, 12]. Finally, changes in CBF during the follicular and luteal phases of the menstrual cycle have recently been demonstrated [49]. However, elucidation of these effects would require a much larger cohort size than in the present study.

Sex differences in CBF may provide insights into their potential role in neurodegenerative diseases, especially given the emerging data regarding the higher incidence rate of Alzheimer’s disease (AD) in women [50–52]. Evidence implicates decreases in regional CBF with subsequent decreased metabolic activity in AD and other forms of dementia [53–57]. These studies and our current findings motivate further investigation of the underlying mechanisms of CBF decline and its role in the development of cognitive impairment, including dementia. If confirmed with longitudinal studies, this could establish maintenance of CBF through pharmacologic or lifestyle interventions as a therapeutic target for prevention of dementia; this could be tailored differently for men and women.

Finally, we note that our measured CBF values are somewhat different from those reported in the literature; this may be attributed to the dependence of such values on ASL sequence type, labeling pulse duration, post-labeling duration, background suppression method, repetition time, echo time, and other factors [33]. Indeed, derived CBF values span a wide age range in the literature, between 40 to 80 mL/min/100g for GM and 10 to 30 mL/min/100g in WM [5–14, 16, 58–60]. Nevertheless, we have employed a self-consistent MRI protocol throughout this study, so that the age and sex related results within our cohort are reliable.

### Limitations

Although our work examines a relatively large cohort and uses advanced MR methodology, certain limitations remain. Our dataset is cross-sectional, so that the CBF associations with age observed here require further validation through longitudinal studies. Such work, motivated by the present results, is underway. Furthermore, we used identical ASL experimental parameters for all subjects and acquired perfusion images at a single post-labeling delay (PLD), implicitly assuming minimal effects from potential spatial variation in arterial transit time (ATT), the transit time of the arterial bolus from the labeling plane to the imaging voxels [33, 61]. Although this is a reasonable assumption [4], ATT may vary spatially within a single subject, and may differ between subjects secondary to arterial blood velocity differences. Further work may implement ASL techniques employing multiple post-labeling delays to address this [33]. In addition, a PLD of 1800 ms was recommended for patients less than 70 yrs. [4]. We fixed PLD to 2000 ms based on the recommendations of Alsop and colleagues: “… a PLD of 2000 ms recommended for the clinical adult population, independent of age, given the potential for a wide variety of pathologies, which are often not known in advance of imaging” [4]. Moreover, as conventional [4], we assumed constant longitudinal and transverse relaxation times for all participants, although these parameters follow complex patterns with aging [30, 62, 63]. Further, although our assessment of white matter hyperintensities (WMHI) did not reveal WMHI in any of the participants of our study cohort, our inspection was based on the PD images only. A thorough evaluation using FLAIR-based images could have provided a better assessment of WMHI. Finally, other factors, including cortical tissue atrophy [5, 64, 65], medications, and dietary intake were not considered in this work.

In conclusion, we examined regional CBF in cerebral WM and GM structures in a cohort of cognitively unimpaired participants across a wide age range. We found lower CBF values with age in GM regions, while higher CBF values with age in WM regions. In addition, women exhibited overall higher CBF values as compared to men. This work may lay the foundation for longitudinal investigations to establish the nature of regional CBF changes with normal aging and neurodegeneration, including Alzheimer’s disease.

## MATERIALS AND METHODS

### Participants

**Figure 5:**
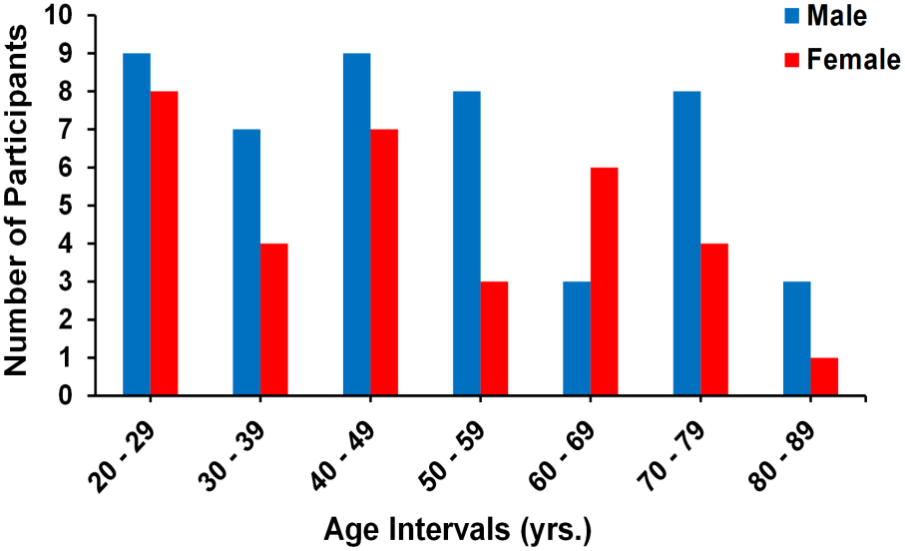
**Number of participants per age decade and sex within the study cohort.**

Investigation has been conducted in accordance with the ethical standards and according to the Declaration of Helsinki and according to national and international guidelines and has been approved by the authors' institutional review board. Participants were drawn from two ongoing healthy aging cohorts at the National Institute on Aging (NIA). 15 volunteers from the Baltimore Longitudinal Study of Aging (BLSA) [66, 67], and 52 from the Genetic and Epigenetic Signatures of Translational Aging Laboratory Testing (GESTALT) were enrolled in this study. Thirteen additional participants from our home institution were recruited. The study populations, experimental design, and measurement protocols of the BLSA have been previously reported [66, 67]. The BLSA is a longitudinal cohort study funded and conducted by the NIA Intramural Research Program (IRP). Established in 1958, the BLSA enrolls community-dwelling adults with no major chronic conditions or functional impairments. The GESTALT study is also a study of healthy volunteers, initiated in 2015, and funded and conducted by the NIA IRP. The goal of the BLSA and GESTALT studies is to evaluate multiple biomarkers related to aging. We note that the inclusion and exclusion criteria for these two studies are essentially identical. Participants underwent testing at the NIA's clinical research unit and were excluded if they had metallic implants, or neurologic or medical disorders. Participants underwent a Mini Mental State Examination (MMSE) and achieved a score > 25. The final cohort consisted of 80 cognitively unimpaired volunteers ranging in age from 22 to 88 years (mean ± standard deviation 49.2 ± 18.7 years) of which 47 were men (49.7 ± 19.2 years) and 33 were women (48.4 ± 18.3 years). Data from four additional subjects were not included due to excessive motion artifacts. The number of participants per age-decade was: 17 (8 women) within 20-29 years, 11 (4 women) within 30-39 years, 16 (7 women) within 40-49 years, 11 (3 women) within 50-59 years, 9 (6 women) within 60-69 years, 12 (4 women) within 70-79 years, and 4 (1 woman) within 80-89 years. Figure 5 provides a detailed distribution of the number of participants per age-decade and sex. Experimental procedures were performed in compliance with our local Institutional Review Board, and participants provided written informed consent.

### Data acquisition

MRI scans were performed on a 3T whole body Philips MRI system (Achieva, Best, The Netherlands) using the internal quadrature body coil for transmission and an eight-channel phased-array head coil for reception. For each participant, multi-slice single shot 2D echo-planar imaging (EPI) pCASL imaging datasets were acquired following the consensus recommendations of the ISMRM Perfusion Study Group and the European ASL in Dementia consortium [4]. This consisted of control, labeled, and proton density (PD) images acquired with incorporation of background suppression, FoV of 220 mm × 210 mm × 120 mm, and spatial resolution of 2.5 mm × 2.5 mm × 5 mm with reconstruction to 1 mm × 1 mm × 1 mm through linear interpolation after scanner reconstruction. 24 slices were acquired in ascending order to avoid slice ordering confounds associated with interleaved order schemes, and with minimal temporal slice spacing to ensure similar post-labeling duration (PLD) for all slices. Other experimental parameters were: echo time (TE) of 15 ms, repetition time (TR) of 7.5 s, labeling duration of 1.8 s, PLD of 2 s, SENSE factor of 2.3, flip angle of 90°, label distance of 8.5 cm, and 30 signal averages [4]. We note that the PD image was acquired with identical TE and TR as for the control and labeled images. The total acquisition time was ~12 min.

### Data processing

After careful visual inspection of data quality for each participant [4], a whole-brain CBF map was generated from the corresponding pCASL dataset using NESMA noise filtering to improve accuracy and precision in CBF determination [15]. Briefly, NESMA-ASL restores the amplitude of an index voxel by incorporating the intensities of voxels with similar multispectral signal patterns, that is, intensities from pCASL images. The similarity between two voxels across the pCASL images is calculated using the relative Euclidean distance within a large search window centered on the index voxel. The size of the search window must be sufficiently large to ensure inclusion of an adequate number of similar voxels, and sufficiently restricted to ensure that the transmission and reception radiofrequency fields and noise standard deviation are approximately constant within the window. Voxels exhibiting relative Euclidean distance lower than 5% are considered as being similar to the index voxel [15]. Finally, CBF maps derived from unfiltered ASL images were also generated for comparison with those derived from the NESMA-filtered ASL images. All CBF maps were calculated based on the following equation [4].

$$\text{CBF}=6000\times \frac{\lambda \;\cdot \;\left(I_{C}-I_{L}\right)\;\cdot \;exp\left(\text{PLD}/T_{1,Blood}\right)}{2\alpha \;\cdot \;T_{1,Blood}\;\cdot \;I_{PD}\;\cdot \;\left(1-exp\left(-\text{LD}/T_{1,Blood}\right)\right)}\left[\text{mL}/100\text{ g}/\text{min}\right],$$

where *I*_C_, *I*_L_, and *I*_PD_ are the control, labeled, and PD images, respectively. Here, *λ* is the partition coefficient between brain tissue and blood with value set to 0.9 mL/g, *α* is the labeling efficiency with value fixed to 0.85, *T*_1,Blood_ = 1.65 s (at 3T) representing the longitudinal relaxation time of blood, and LD and PLD are the label duration and post labeling delay, respectively.

The PD image of each participant was nonlinearly registered to the Montreal Neurological Institute (MNI) space with 1 mm × 1 mm × 1 mm voxel resolution, using FNIRT as implemented in the FMRIB Software library (FSL) [68]. Using FSL, FAST segmentation was performed to generate WM and GM masks. Figure 1 shows examples of GM and WM masks for fourteen participants within different age decades. Six regions of interest (ROIs) were defined from the MNI structural atlas corresponding to the whole brain, and the frontal, parietal, temporal, and occipital lobes, and the cerebellum. In each ROI, only voxels with at least 90% of GM or WM, as defined from the FSL-FAST WM and GM masks, were considered to minimize partial volume effects. The mean CBF value within each ROI was then calculated.

### Statistical analysis

For each ROI, the effects of age and sex on CBF were investigated using multiple linear regression with the mean CBF value within each WM or GM ROI as the dependent variable and age and sex as the independent variables. The initial model incorporated an interaction term between sex and age which was removed if found not to be significant. The resulting parsimonious model was then constructed without this interaction term. This analysis was conducted on CBF values derived both with and without use of NESMA-ASL filtering to define the possible effect of filtering on our results.

Further, the effect of age on CBF for men and for women separately was also investigated with the mean CBF value within each WM or GM ROI as the dependent variable and age as the independent variable. For all statistical analyses, the threshold for statistical significance was p < 0.05 after correction for multiple ROI comparisons (*i.e.* 12 ROIs) using the false discovery rate (FDR) method [69, 70]. All calculations were performed with MATLAB (MathWorks, Natick, MA, USA).
